# FOLFIRI plus cetuximab or bevacizumab for advanced colorectal cancer: final survival and per-protocol analysis of FIRE-3, a randomised clinical trial

**DOI:** 10.1038/s41416-020-01140-9

**Published:** 2020-11-06

**Authors:** Volker Heinemann, Ludwig Fischer von Weikersthal, Thomas Decker, Alexander Kiani, Florian Kaiser, Salah-Edin Al-Batran, Tobias Heintges, Christoph Lerchenmüller, Christoph Kahl, Gernot Seipelt, Frank Kullmann, Markus Moehler, Werner Scheithauer, Swantje Held, Lisa Miller-Phillips, Dominik Paul Modest, Andreas Jung, Thomas Kirchner, Sebastian Stintzing

**Affiliations:** 1 Department of Medicine III, University Hospital, Ludwig Maximilian University (LMU), Munich, Germany; 2 Gesundheitszentrum St. Marien, Amberg, Germany; 3 Onkologie Ravensburg, Ravensburg, Germany; 4 Klinik Herzoghöhe, Bayreuth, Germany; 5 Oncological Practice, Landshut, Germany; 6 Institute of Clinical Cancer Research at Krankenhaus Nordwest University Cancer Center, Frankfurt, Germany; 7 Department of Medicine II, Städtische Kliniken Neuss, Neuss, Germany; 8 Oncological Practice, Münster, Germany; 9 Department of Haematology and Oncology, Städtisches Klinikum Magdeburg, Magdeburg, Germany; 10 Oncological Practice, Bad Soden, Germany; 11 Department of Medicine I, Klinikum Weiden, Weiden, Germany; 12 Department of Medicine II, University Hospital, Johannes Gutenberg University Mainz, Mainz, Germany; 13 Department of Internal Medicine I and Comprehensive Cancer Center, Medical University of Vienna, Vienna, Austria; 14 ClinAssess GmbH, Leverkusen, Germany; 15 Institute of Pathology, University of Munich, Munich, Germany; 16 Division of Hematology, Oncology, and Tumor Immunology (CCM), Department of Medicine, Charité Universitaetsmedizin Berlin, Berlin, Germany

**Keywords:** Oncology, Biomarkers

## Abstract

**Background:**

Cetuximab plus FOLFIRI improved overall survival compared with bevacizumab plus FOLFIRI in *KRAS* wild-type metastatic colorectal cancer (mCRC) in FIRE-3, but no corresponding benefit was found for progression-free survival. This analysis aimed to determine whether cetuximab improves response and survival versus bevacizumab among response-evaluable patients receiving first-line FOLFIRI for *RAS* wild-type mCRC and the effect of primary tumour side on outcomes.

**Methods:**

The intent-to-treat population included 593 patients with *KRAS* exon 2 wild-type mCRC. Further testing identified 400 patients with extended *RAS* wild-type disease; of these, 352 (88%) who received ≥3 cycles of therapy and had ≥1 post-baseline scan were evaluable for response and constituted the per-protocol population (169 cetuximab and 183 bevacizumab). Patients received 5-fluorouracil, folinic acid and irinotecan (FOLFIRI) with either weekly cetuximab or biweekly bevacizumab given on day 1 of each 14-day cycle until response, progression or toxicity occurred. The primary endpoint was the objective response rate (ORR) in the per-protocol population. Secondary endpoints included overall survival (OS) and progression-free survival (PFS). The effect of primary tumour location was evaluated.

**Results:**

Median OS in the *RAS* wild-type population was 31 vs 26 months in the cetuximab and bevacizumab groups, respectively (HR 0.76, *P* = 0.012). In the per-protocol population, outcomes favoured cetuximab for ORR (77% vs 65%, *P* = 0.014) and median OS (33 vs 26 months, HR 0.75, *P* = 0.011), while PFS was comparable between groups. The advantage of cetuximab over bevacizumab occurred only in patients with left-sided primary tumours.

**Conclusions:**

FOLFIRI plus cetuximab resulted in a significantly higher ORR and longer OS compared to FOLFIRI plus bevacizumab among patients with left-sided tumours. The superior response associated with cetuximab may particularly benefit patients with symptomatic tumours or borderline-resectable metastases.

**ClinicalTrials.gov identifier:**

NCT00433927.

## Background

Median survival of 30 months or more is now achievable for patients with metastatic colorectal cancer (mCRC) who receive multimodal treatment as part of a continuum of care incorporating first-line and subsequent therapies.^1^ 5-Fluorouracil-based chemotherapy combined with a biological agent is recommended as initial therapy for most patients, while selection of the optimum combination and sequencing depends on upfront molecular profiling of the tumour.^1,2^

The FIRE-3 study (AIO KRK-0306) was a randomised, open-label, phase 3 trial that compared the efficacy of cetuximab and bevacizumab when added to first-line 5-fluorouracil, folinic acid and irinotecan (FOLFIRI) in patients with *KRAS* exon 2 wild-type mCRC. During the study, the importance of additional *RAS* mutations in determining the efficacy of epidermal growth factor receptor (EGFR) inhibitors became apparent, with extended *RAS* mutation testing now mandatory before administering these agents.^1,2^ Accordingly, a *RAS* wild-type subset of patients in FIRE-3 was identified and corresponds to the currently licensed population for cetuximab. As previously reported, overall survival (OS) was significantly longer with FOLFIRI plus cetuximab when compared to FOLFIRI plus bevacizumab, both in the intention-to-treat (ITT) population of patients with *KRAS* exon 2 wild-type disease, and in the final *RAS* wild-type population.^3,4^

This report presents a final survival update of FIRE-3 and evaluates the response rate in the per-protocol population, consisting of all patients with *RAS* wild-type disease who received three or more cycles of therapy and had at least one radiological evaluation post baseline. The effect of primary tumour side on outcomes is also investigated.

## Methods

Details of the FIRE-3 study design, patient-eligibility criteria, randomisation and treatment have been reported previously, and the trial protocol is available in Supplementary information 1.^4^ The trial was performed in accordance with the Declaration of Helsinki, with ethics committee approval from all participating centres, and written informed consent from all patients. Patients recruited during 2007–2012 at 110 German and six Austrian centres were centrally randomised in a 1:1 ratio with stratification by Eastern Cooperative Oncology Group (ECOG) performance status, number of metastatic sites, white blood cell count and alkaline phosphatase levels. Randomisation of 568 patients was calculated to provide a power of 80% to detect a response rate of 62 vs 50% in favour of the FOLFIRI plus cetuximab arm with a one-sided Fisher’s exact test significance level of 2.5%.^4^

### *RAS* mutation status

In October 2008, the protocol was amended to restrict entry to patients without *KRAS* exon 2 mutations. Of 752 patients who provided informed consent, 593 had *KRAS* exon 2 wild-type disease and began treatment; this was defined as the ITT population. In a post hoc analysis, extended *RAS* testing was successfully performed in tumour samples from 475 patients to determine mutation status in *KRAS* and *NRAS* exons 2–4.^3,4^ The extended *RAS* sequencing was performed in a certified, quality-assured laboratory (Institute of Pathology, LMU, Munich) and identified 400 patients with no mutations in the *RAS* genes tested; this was defined as the *RAS* wild-type population.^3^

### Study treatment

Cetuximab or bevacizumab was administered with FOLFIRI on day 1 of each 14-day treatment cycle.^4^ The initial cetuximab dose was 400 mg/m^2^, then 250 mg/m² weekly, the bevacizumab dosage was 5 mg/kg of bodyweight every 2 weeks and FOLFIRI consisted of 5-fluorouracil 400 mg/m^2^ (IV bolus), folinic acid 400 mg/m^2^ and irinotecan 180 mg/m^2^, followed by a continuous 46-h infusion of fluorouracil 2400 mg/m^2^. In the absence of disease progression or unacceptable toxicity, treatment was continued until complete response or conversion to surgical resectability was attained, or the patient or physician decided to discontinue treatment. Tumour response, study endpoints and per-protocol analysis was performed.

The primary endpoint in FIRE-3 was the investigator-assessed objective response rate (ORR, complete or partial response) according to the Response Evaluation Criteria in Solid Tumours (RECIST) criteria, version 1.0.^5^ The first CT scan was performed after 3 cycles of therapy; thus, the per-protocol analysis included all *RAS* wild-type patients who received ≥3 cycles and had at ≥1 post-baseline CT scan allowing evaluation of response. Secondary endpoints included progression-free survival (PFS), OS, secondary resection of liver metastases with curative intent and safety. Adverse events were recorded according to the National Cancer Institute Common Terminology Criteria for Adverse Events (CTCAE), version 3.0.

Early tumour shrinkage, defined as a reduction of ≥20% in the sum of the longest tumour diameters of the selected target lesions at week 6, and depth of response, defined as the maximum percentage change in tumour size compared to baseline, were analysed using data from an independent, centralised radiological review of response using RECIST 1.1 criteria.^6^ The independent review was performed by board-certified radiologists (Radiology Consulting GmbH, Leverkusen, Germany) who were masked to treatment allocation, and included all 332 patients (*n* = 332) with *RAS* wild-type disease for whom pre- and post-treatment CT scans were available.^3^

### Primary tumour location

The effect of primary tumour location (right- or left-sided) on outcomes was retrospectively assessed in the *RAS* wild-type population, where right-sided refers to tumours located in the caecum to the transverse colon and left-sided to tumours from the splenic flexure to the rectum.

### Statistical analysis

The study ended in November 2017 and the database was closed in March 2018. Statistical evaluation was performed by ClinAssess GmbH according to the statistical analysis plan using SAS^®^ version 9.4. Tumour-response rates, including objective response and early tumour shrinkage, were compared between groups using a two-sided Fisher’s exact test (α = 0.05), while a two-sided Wilcoxon test was used to compare the depth of tumour response. The duration of follow-up was analysed using the inverse Kaplan–Meier method. Survival, including rates at specific times, was analysed using the Kaplan–Meier method and compared with log-rank tests, while hazard ratios were estimated using a Cox proportional hazard regression model. All comparisons between groups were done with a two-sided test and α = 0.05; no adjustment of α was done. Data on sidedness of the primary are of retrospective nature and no adjustment for multiple testing has been performed.

## Results

### Disposition of the *RAS* wild-type and per-protocol populations

Supplementary Fig. S1 shows the disposition of patients in the *RAS* wild-type and per-protocol populations. Of 400 patients in the *RAS* wild-type population, 48 were not evaluable for response: 29 received fewer than 3 cycles of chemotherapy (see below) and 19 had no post-baseline radiographic evaluation available. The remaining 352 (88%) patients constituted the per-protocol population, including 169 who received FOLFIRI plus cetuximab and 183 who received FOLFIRI plus bevacizumab.

The proportion of patients excluded from the per-protocol analysis was higher in the cetuximab group (30/199, 15%) compared with the bevacizumab group (18/201, 9%). Supplementary Table S1 summarises the reasons for exclusion: most commonly, allergic reaction (nine patients in the cetuximab group vs zero in the bevacizumab group), patient decision (four in each group), early death and thromboembolic event or bleeding (both recorded for one and four patients in the cetuximab and bevacizumab groups, respectively).

Patient characteristics were highly similar in the *RAS* wild-type and per-protocol populations and were generally comparable between the two treatment arms at baseline (Supplementary Table S2). Patients were aged 31–76 years, with a median age of 64–65 years in each treatment arm. The proportion of females in the per-protocol population was 24% in the FOLFIRI plus cetuximab arm and 34% in the FOLFIRI plus bevacizumab arm. Most patients had left-sided primary tumours; in the per-protocol population, 18% of patients in the cetuximab arm and 25% in the bevacizumab arm had right-sided primary tumours.

### Safety and tolerability

Adverse events have been reported in detail for the ITT population.^4^ The safety population for this analysis consisted of all 400 patients with *RAS* wild-type tumours. Treatment-related adverse events occurred in all but two patients; these were grade 3 or higher in 127 (64%) of FOLFIRI plus cetuximab and 103 (51%) of FOLFIRI plus bevacizumab recipients. Overall, 46 (12%) of patients were withdrawn due to treatment-related adverse events (15% in the cetuximab arm and 8% in the bevacizumab arm). Adverse events were consistent with the known toxicity profiles of each agent (see Supplementary Table S3).

Serious adverse events (SAEs) related to study medication occurred in 33 (17%) of FOLFIRI plus cetuximab and 38 (19%) of FOLFIRI plus bevacizumab recipients. Five deaths associated with SAEs were recorded in the FOLFIRI plus bevacizumab group, including three deaths (1.5%) that were considered treatment-related. There were no deaths from adverse events in the cetuximab group.

### Efficacy in the *RAS* wild-type population

**Fig. 1:**
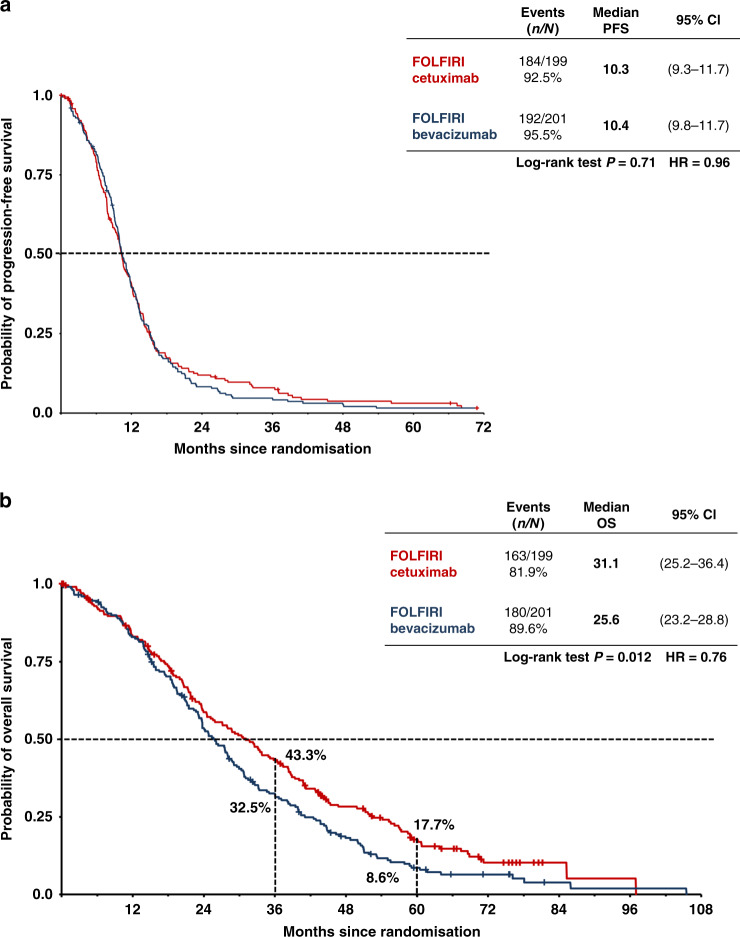
Survival times in the RASwt population (*n* = 400). Progression-free (**a**) and overall (**b**) survival in the *RAS* wild-type population (*N* = 400). Bev bevacizumab, Cet cetuximab.

The median follow-up in the overall *RAS* wild-type population was 71 months (95% CI, 66–77) and 76 months (95% CI, 66 to not reached) in the cetuximab and bevacizumab groups, respectively. At the time of analysis, 82% of patients in the FOLFIRI plus cetuximab arm and 90% of patients in the FOLFIRI plus bevacizumab arm had died. OS was significantly better in the cetuximab group vs the bevacizumab group (*P* = 0.012), with estimated 3-year and 5-year survival rates of 43 vs 33% and 18 vs 9%, respectively (Fig. 1). Median OS was 31 months (95% CI, 25–36) in the cetuximab group and 26 months (95% CI, 23–29) in the bevacizumab group (hazard ratio [HR] 0.76, 95% CI, 0.62–0.94) (Supplementary Table S4). This survival benefit was limited to patients with left-sided primary tumours (*n* = 307, HR 0.70, *P* = 0.004), with no significant difference between groups in patients with right-sided primary tumours (*n* = 88, HR 1.27, *P* = 0.29) (Supplementary Table S4 and Supplementary Fig. S2).

Neither the ORR (66% vs 59% in the cetuximab and bevacizumab groups, respectively) nor PFS (median 10.3 vs 10.4 months) differed significantly between treatment groups, although patients in the cetuximab group were more likely to experience early tumour shrinkage and had a significantly greater median depth of tumour response (Supplementary Table S4). The disease control rate (response or stable disease as the best overall response) was 81% in the cetuximab group and 87% in the bevacizumab group, while the rate of disease progression was 4.5% in both groups (Supplementary Table S5). With respect to secondary resectability, 40 patients in the cetuximab arm (20.1%) and 47 patients (23.4%) in the bevacizumab arm underwent secondary metastasectomy in curative intent. The relapse rate after resection in curative intent as the reason for termination of the study treatment was 64% in FOLFIRI plus cetuximab-treated patients and 80% in FOLFIRI plus bevacizumab-treated patients. Median times to relapse after resection were 7.0 months in the cetuximab arm and 7.4 months in the bevacizumab arm (HR 0.81; log-rank *p* = 0.53). Post-resection OS was 52.0 months and 33.7 months, respectively (HR 0.65; log-rank *p* = 0.24). Data on the influence of CMS on early tumour shrinkage can be found in Supplementary Table S6.

### *RAS* wild-type per-protocol analysis

**Fig. 2:**
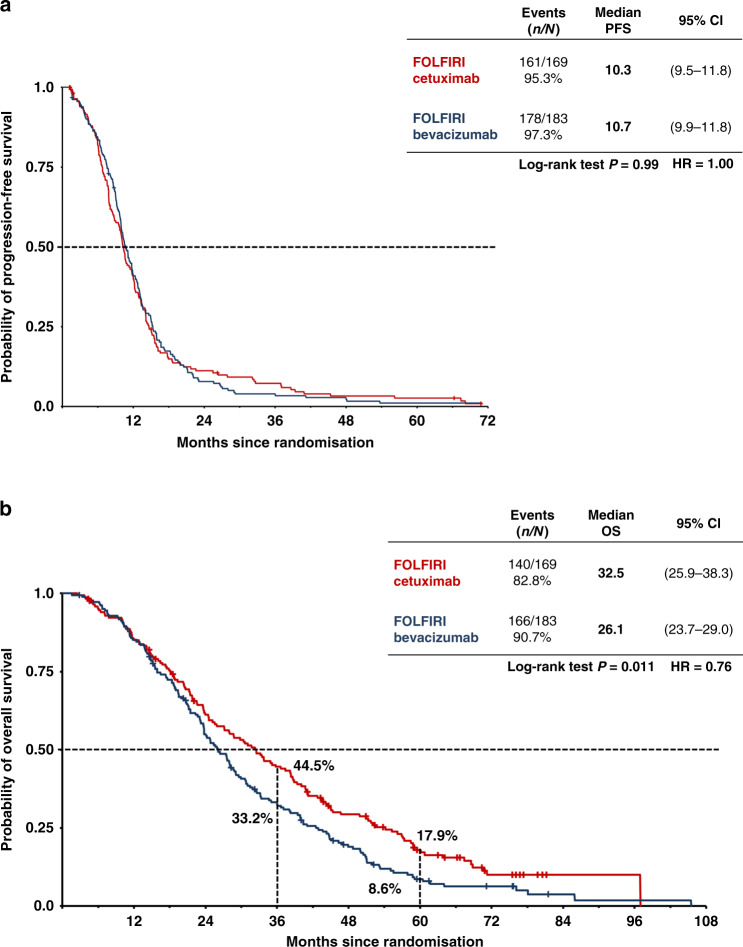
Survival times in the RASwt population that were per protocol accessible for tumour response (*n* = 352). Progression-free (**a**) and overall (**b**) survival in the *RAS* wild-type per-protocol population (*N* = 352). Bev bevacizumab, Cet cetuximab.

**Fig. 3:**
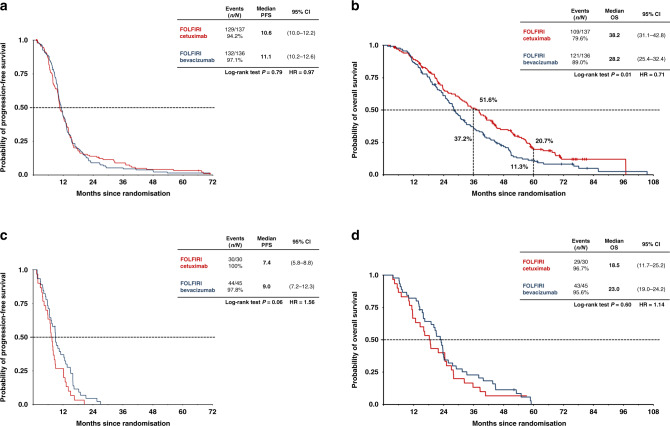
Primary tumour location and survival in the *RAS* wild-type per-protocol population. Left-sided tumours (*N* = 273): **a** progression-free survival, **b** overall survival; right-sided tumours (*N* = 75): **c** progression-free survival, **d** overall survival. Bev bevacizumab, Cet cetuximab.

Table 1 summarises efficacy in the *RAS* wild-type per-protocol population. The ORR was significantly higher in the FOLFIRI plus cetuximab group (77 vs 65%), whereas there was no difference in the disease control rate (95% in both groups, Supplementary Table S5) or PFS (Table 1 and Fig. 2a). OS was superior in the cetuximab group, with median survival of 33 vs 26 months in patients treated with FOLFIRI plus cetuximab and FOLFIRI plus bevacizumab, respectively (Table 1 and Fig. 2b). However, these outcomes differed by primary tumour location. In both treatment groups, efficacy was consistently lower in patients with right-sided vs left-sided primary tumours (Table 1 and Fig. 3). Furthermore, the significant advantage in the cetuximab group for response and OS was observed only in patients with left-sided tumours. The OS rates in this subset were 52% for FOLFIRI plus cetuximab and 37% for FOLFIRI plus bevacizumab at 3 years, and 21 vs 11% at 5 years (Fig. 3b); the corresponding values for patients with right-sided tumours were 13 vs 23% at 3 years and 7% vs 0 at 5 years (Fig. 3d). In contrast, PFS did not differ between treatment groups in patients with left-sided tumours (Fig. 3a), while among patients with right-sided tumours, there was a trend towards longer PFS in the bevacizumab group (HR 1.56, *P* = 0.06) (Fig. 3c).

**Table 1:** Efficacy in the *RAS* wild-type per-protocol population (*N* = 352)

	FOLFIRI plus cetuximab (*N* = 169)	FOLFIRI plus bevacizumab (*N* = 183)	OR/HR (95% CI)	*P* value
*All patients*				
ORR, *n* (%) [95% CI]	130 (77) [70–83]	118 (65) [57–71]	1.84^b^ (1.15–2.93)	**0.014**
Early tumour shrinkage, *n* (%) (*n* = 320)	105/150^a^ (70)	85/170^a^ (50)	2.33^b^ (1.47–3.70)	**0.0004**
Median depth of response, % (*n* = 320)^a^	50	33	NA	**<0.0001**
Median PFS, months (95% CI)	10 (10–12)	11 (10–12)	0.99^**c**^ (0.81–1.24)	1.00
Median OS, months (95% CI)	33 (26–38)	26 (24–29)	0.75^**c**^ (0.59–0.94)	**0.011**
*Left-sided primary tumours*	(*N* = 137)	(*N* = 136)		
ORR, n (%) [95% CI]	108 (79) (71–85)	92 (68) (59–75)	1.78^b^ (1.03–3.07)	**0.041**
Median PFS, months (95% CI)	11 (10–12)	11 (10–13)	0.97^c^ (0.76–1.24)	0.79
Median OS, months (95% CI)	38 (31–43)	28 (25–32)	0.71^c^ (0.55–0.92)	**0.01**
*Right-sided primary tumours*	(*N* = 30)	(*N* = 45)		
ORR, *n* (%) [95% CI]	20 (67) (47–83)	25 (56) (40–70)	1.60^b^ (0.61–4.18)	0.47
Median PFS, months (95% CI)	7 (6–9)	9 (7–12)	1.56^c^ (0.97–2.52)	0.06
Median OS, months (95% CI)	19 (12–25)	23 (19–24)	1.14^c^ (0.71–1.84)	0.60

*CI* confidence interval, *FOLFIRI* fluorouracil, folinic acid and irinotecan, *HR* hazard ratio, *NA* not applicable, *OR* odds ratio, *ORR* objective response rate, *OS* overall survival, *PFS* progression-free survival.

^a^Early tumour shrinkage and depth of response were centrally reviewed for all patients with available data.

^b^Odds ratio.

^c^Hazard ratio.

### Intention-to-treat population: final results

The median follow-up duration in the ITT population of 593 patients with *KRAS* exon 2 wild-type disease was 70–71 months in both groups. Median OS was 28 months (95% CI, 24–32) in the FOLFIRI plus cetuximab arm and 26 months (95% CI, 23–28) in the FOLFIRI plus bevacizumab arm (HR 0.84, *P* = 0.051), with a 5-year survival rate of 16 vs 9%. A list of baseline characteristics of patients surviving in 3, 4 and 5 years is found in the supplement (Supplementary Table S7). The centrally reviewed response rate was significantly higher in the cetuximab arm (ORR 67%; 95% CI, 60–73%) compared with the bevacizumab arm (ORR 55%; 95% CI, 49–61%), with an odds ratio of 1.62 (*P* = 0.01). However, median PFS was similar in both groups (10.1 vs 10.5 months; HR 1.06; *P* = 0.46).

## Discussion

The final OS analysis of FIRE-3 confirms the benefit of first-line treatment with FOLFIRI plus cetuximab when compared to FOLFIRI plus bevacizumab in mCRC patients with *RAS* wild-type tumours. After a median follow-up of almost 6 years, the observed OS times clearly favoured the cetuximab arm (HR 0.75, *P* = 0.011 in the per-protocol population). This result is highly consistent with the previously published data from the PEAK study comparing FOLFOX plus panitumumab to FOLFOX plus bevacizumab and is further supported by a meta-analysis of data from the three available head-to-head trials, which also included the CALGB 80405 study.^7,8^ With regard to toxicity, no new or unexpected toxicities were observed, and adverse events of grade 3 or higher were in accordance with previous reports.^9,10^ Due to expected toxicities of cetuximab, such as anaphylactic reactions, the number of patients unevaluable according to the protocol was higher in the cetuximab arm (*n* = 30) than in the bevacizumab arm (*n* = 18). Especially skin toxicities have to be discussed with the patient before cetuximab is started to ensure compliance.

The per-protocol population of FIRE-3 included only patients who received at least three cycles of study medication and had a second radiologic evaluation after baseline. In this cohort, for which the primary endpoint of investigator-assessed ORR was evaluable according to RECIST, the ORR was significantly higher with FOLFIRI plus cetuximab compared to FOLFIRI plus bevacizumab in all patients (77 vs 65%, *P* = 0.014) and patients with left-sided tumours (79 vs 68%, *P* = 0.041), but not in the smaller subset of patients with right-sided tumours (67 vs 56%, *P* = 0.47). This benefit was also supported by a significantly higher rate of patients obtaining early tumour shrinkage (70 vs 50%, *P* = 0.0004), together with a significantly greater depth of response (50 vs 33%, *P* < 0.0001). For patients in need of tumour response due to symptomatic tumours or borderline-resectable metastases, anti-EGFR treatment, irrespective of the tumour location, provides the fastest and most extensive tumour response when compared to either chemotherapy alone or chemotherapy plus bevacizumab.^9,11–13^ Although PFS was comparable in both treatment arms of FIRE-3, superior ORR in the cetuximab arm was associated with a significantly longer post-progression survival, which probably resulted in the observed differences in OS.^14^ Within the trials investigating anti-EGFR treatment vs anti-vascular endothelial growth factor (VEGF) treatment in the first-line treatment of mCRC patients, only the PEAK study showed a significant difference in PFS favouring the anti-EGFR arm.^15^ The reasons for this observation are not yet understood. Data with respect to the best sequence strategy in RASwt mCRC are scarce. Several retrospective analyses have shown a favourable outcome for the use of EGFR antibodies followed by bevacizumab than the other way round.^16,17^ Preclinical data suggest an EGFR-independent activation of the MAPK pathway by STAT3 and ERK modulated through VEGFR2 activation by higher VEGF levels.^18^ As higher VEGF levels are seen in patients pre-treated with bevacizumab, this effect may contribute to the observed clinical outcome data. In both arms, the number of patients who underwent surgery in curative intent was comparable, so secondary resectability is not the reason for the observed OS difference. The observed OS difference may therefore be attributed to the more pronounced tumour response, reflected by the depth-of-response data, in combination with the more favourable treatment sequence of anti-EGFR treatment followed by anti-VEGF than the other way round.^16^

Evaluation of outcomes according to sidedness demonstrated that the OS benefit was limited to patients with left-sided primary tumours. In this subgroup, OS clearly favoured the cetuximab arm, both in the final *RAS* wild-type population (HR 0.70, *P* = 0.004) and in patients who were treated according to the protocol (HR 0.71, *P* = 0.01); the median OS benefit over the bevacizumab arm reached 8 and 10 months, respectively (Supplementary Table A.4 and Table 1). This led to markedly superior 3- and 5-year survival rates for FOLFIRI plus cetuximab recipients. Indeed, 5-year survival rates were almost doubled, reaching 21% for left-sided tumours treated using FOLFIRI plus cetuximab, compared to 11% using FOLFIRI plus bevacizumab (Fig. 3b). The benefit of anti-EGFR treatment for *RAS* wild-type, left-sided tumours has also been demonstrated in the CALGB 80405 study.^19,20^

While the superior ORR induced by the addition of cetuximab to FOLFIRI translated into longer OS in patients with left-sided primaries, this was not the case in patients with right-sided primary tumours, where numerically higher response rates did not lead to longer PFS or OS. In fact, PFS (HR 1.56, *P* = 0.06) and OS (HR 1.14, *P* = 0.60) favoured bevacizumab treatment in right-sided tumours, without, however, reaching the level of statistical significance.

Although the analysis by tumour sidedness was limited by its retrospective nature and the rather small number of patients with right-sided tumours, data were shown to be highly consistent across different studies. Specifically, the predictive effect of sidedness with regard to the efficacy of EGFR-targeted antibodies has been supported by several analyses.^12,21^ Consequently, recent treatment recommendations have included sidedness as a relevant factor to guide decision-making in patients with *RAS* wild-type tumours.^2,13,22^

While the addition of anti-EGFR agents to chemotherapy clearly improves tumour response, it is hypothesised that development of resistance may be faster in right-sided tumours, resulting in shorter PFS and post-progression survival. Therefore, anti-EGFR treatment may be considered in right-sided tumours if induction of tumour response for conversion therapy is the primary goal;^22^ to avoid a negative impact on OS, this approach requires an immediate switch of therapy in patients not responding to induction treatment. If OS is the primary goal in *RAS* wild-type right-sided tumours with a widely disseminated disease not amenable to surgery, anti-EGFR substances may be discussed but are not the primary choice.

## Conclusion

The final update of FIRE-3 confirms the previously reported superiority of FOLFIRI plus cetuximab when compared to FOLFIRI plus bevacizumab. Combining a molecular marker (*RAS* mutational analysis) with a clinical characteristic (tumour sidedness), the patient population most likely to benefit from anti-EGFR strategy can be defined. For patients with left-sided *RAS* wild-type primaries, this personalised approach promises high tumour-response rates, together with a meaningfully prolonged OS, a doubling of long-term survival rates and overall manageable toxicities.

## Supplementary information

Supplementary Data Set

## Data Availability

All authors had access to the data published in this paper. Data have been uploaded to the Pharmnet.bund online platform of the German federal department of health (https://portal.dimdi.de/data/ctr/O-0329_01-2-1-B80630-20190731152224.pdf).
