# Supplementary material for: FOLFIRI plus cetuximab or bevacizumab for advanced colorectal cancer: final survival and per-protocol analysis of FIRE-3, a randomised clinical trial
Source: Br J Cancer. 2020 Nov 6;124(3):587–94. doi: 10.1038/s41416-020-01140-9 (PMC7851157; doi:10.1038/s41416-020-01140-9)

# Supplementary Appendix. A

The statistical analysis plan and protocol for the FIRE-3 study are available online.

## **Table S.1. Reasons for exclusion of patients from the per-protocol analysis, by treatment group**

| **Reason for exclusion** | **FOLFIRI plus  cetuximab (*N*=30)** | **FOLFIRI plus  bevacizumab (*N*=18)** |
| --- | --- | --- |
| Allergic reaction | 9 |  |
| Clinical progression | 1 |  |
| Deterioration of performance status | 1 |  |
| Diarrhoea | 1 | 2 |
| Early death | 1 | 4 |
| Elevated liver enzymes | 1 |  |
| Hypotension | 1 |  |
| Ileus | 2 | 1 |
| Ineligible (inclusion criteria violation) |  | 1 |
| Infectious complication | 3 |  |
| Patient wish / withdrawal of consent | 4 / 0 | 3 / 1 |
| Radiation therapy | 1 |  |
| Resectability |  | 1 |
| Skin toxicity | 2 |  |
| Thromboembolic event / bleeding | 1 / 0 | 3 / 1 |
| Treatment pause >3 weeks | 2 | 1 |

## **Table S.2. Characteristics of the *RAS* wild-type (N=400) and per-protocol (N=352) populations**

|  | ***RAS* wild-type population** | | **Per-protocol population** | |
| --- | --- | --- | --- | --- |
|  | **FOLFIRI plus cetuximab (*N*=199)** | **FOLFIRI plus bevacizumab (*N*=201)** | **FOLFIRI plus cetuximab (*N*=169)** | **FOLFIRI plus bevacizumab (*N*=183)** |
| Median age, years (range) | 64 (41–76) | 64 (31–76) | 65 (41–76) | 64 (31–76) |
| Age >65 years, *n* (%) | 95 (48) | 96 (48) | 83 (49) | 83 (45) |
| Female, *n* (%) | 52 (26) | 68 (34) | 41 (24) | 62 (34) |
| ECOG performance status:  0  1  2 | 108 (54) 88 (44) 3 (2) | 109 (54) 89 (44) 3 (2) | 94 (56) 73 (43) 2 (1) | 98 (54) 82 (45) 3 (2) |
| Site of primary tumour:  Colon  Rectum  Colon and rectum | 119 (60) 72 (36) 8 (4) | 126 (63) 68 (34) 7 (4) | 99 (59) 63 (37) 7 (4) | 116 (63) 61 (33) 6 (3) |
| Primary tumour side:  Left (hindgut)  Right (midgut)  Both or unknown | 158 (79) 38 (19) 3 (2) | 149 (74) 50 (25) 2 (1) | 137 (81) 30 (18) 3 (2) | 136 (74) 45 (25) 2 (1) |
| No. of metastatic sites:  1  2  ≥3  Unknown | 84 (42) 66 (33) 47 (24) 2 (1) | 82 (41) 69 (34) 49 (24) 2 (1) | 72 (43) 56 (33) 41 (24) 0 (0) | 76 (42) 65 (36) 42 (23) 0 (0) |
| Metastatic sites:  Liver only  Lung only | 71 (36) 5 (3) | 62 (31) 11 (6) | 62 (37) 4 (2) | 58 (32) 10 (6) |
| Prior therapy:  Primary tumour resection  Adjuvant chemotherapy | 168 (84) 37 (19) | 176 (88) 38 (19) | 142 (84) 34 (20) | 163 (89) 34 (19) |

Percentages may not total 100 due to rounding.

## **Table S.3. Adverse events in the *RAS* wild-type population (N=400)**

| **Adverse event** | **FOLFIRI + cetuximab (*N=*199)** | | | | **FOLFIRI + bevacizumab (*N=*201)** | | | |
| --- | --- | --- | --- | --- | --- | --- | --- | --- |
|  | Any grade | | Grade ≥3 | | Any grade | | Grade ≥3 | |
|  | *n* | % | *n* | % | *n* | % | *n* | % |
| **Haematotoxicity** | 181 | 91.0 | 58 | 29.1 | 189 | 94.0 | 46 | 22.9 |
| Leukopenia | 139 | 69.9 | 31 | 15.6 | 136 | 67.7 | 24 | 11.9 |
| Anaemia | 174 | 87.4 | 6 | 3.0 | 188 | 93.5 | 3 | 1.5 |
| Thrombocytopenia | 53 | 26.6 | 1 | 0.5 | 48 | 23.9 | 1 | 0.5 |
| Neutropenia | 127 | 63.8 | 61 | 30.7 | 128 | 63.7 | 44 | 21.9 |
| Febrile Neutropenia | 2 | 1.0 | 2 | 1.0 | 1 | 0.5 | 1 | 0.5 |
| **Non-haematological toxicity** |  |  |  |  |  |  |  |  |
| **Gastrointestinal disorders** | 166 | 83.4 | 35 | 17.6 | 3 | 1.5 |  |  |
| Nausea | 99 | 49.7 | 7 | 3.5 | 132 | 65.7 | 29 | 14.4 |
| Vomiting | 48 | 24.1 | 5 | 2.5 | 69 | 34.3 | 8 | 4.0 |
| Diarrhoea | 121 | 60.8 | 23 | 11.6 | 3 | 1.5 |  |  |
| Mucositis/stomatitis | 85 | 42.7 | 9 | 4.5 | 181 | 90.0 | 47 | 23.4 |
| Obstipation | 52 | 26.1 | 1 | 0.5 | 47 | 23.4 | 3 | 1.5 |
| **Bleeding (respiratory tract, GI, GU)** | 45 | 22.6 | 2 | 1.0 | 60 | 29.9 | 1 | 0.5 |
| **Cardiac disorders** | 15 | 7.5 | 2 | 1.0 | 189 | 94.0 | 46 | 22.9 |
| Arrhythmias | 13 | 6.5 | 1 | 0.5 | 20 | 10.0 | 7 | 3.5 |
| Other | 3 | 1.5 | 2 | 1.0 | 14 | 7.0 | 4 | 2.0 |
| **Endocrine disorders** | 6 | 3.0 | 2 | 1.0 | 6 | 3.0 | 3 | 1.5 |
| **General disorders and administration-site conditions** | 154 | 77.4 | 17 | 8.5 | 166 | 82.6 | 19 | 9.5 |
| Fatigue (asthenia, lethargy) | 104 | 52.3 | 2 | 1.0 | 113 | 56.2 | 3 | 1.5 |
| Fever, without neutropenia grade 3/4 | 35 | 17.6 | 1 | 0.5 | 32 | 15.9 | 1 | 0.5 |
| Medical device complication | 3 | 1.5 | 1 | 0.5 | 2 | 1.0 |  |  |
| Oedema | 31 | 15.6 | 2 | 1.0 | 21 | 10.4 | 1 | 0.5 |
| Pain | 107 | 53.8 | 8 | 4.0 | 119 | 59.2 | 11 | 5.5 |
| Wound healing problems | 4 | 2.0 |  |  | 1 | 0.5 |  |  |
| **Hepatobiliary disorders** | 137 | 68.8 | 15 | 7.5 | 120 | 59.7 | 13 | 6.5 |
| Liver toxicity | 137 | 68.8 | 15 | 7.5 | 120 | 59.7 | 13 | 6.5 |
| **Immune system disorders** | 2 | 1.0 | 1 | 0.5 | 1 | 0.5 |  |  |
| Allergic reaction | 2 | 1.0 | 1 | 0.5 | 1 | 0.5 |  |  |
| **Infections and infestations** | 98 | 49.2 | 23 | 11.6 | 101 | 50.2 | 24 | 11.9 |
| Abscess | 5 | 2.5 | 2 | 1.0 | 5 | 2.5 |  |  |
| Infection, with neutropenia grade 3/4 | 6 | 3.0 | 4 | 2.0 | 13 | 6.5 | 6 | 3.0 |
| Infection, without neutropenia grade 3/4 | 96 | 48.2 | 20 | 10.1 | 97 | 48.3 | 21 | 10.4 |
| **Injury** | 2 | 1.0 | 1 | 0.5 | 3 | 1.5 |  |  |
| Bone fracture | 2 | 1.0 | 1 | 0.5 | 3 | 1.5 |  |  |
| **Metabolism and nutrition disorders** | 129 | 64.8 | 27 | 13.6 | 86 | 42.8 | 22 | 10.9 |
| Electrolyte imbalance | 129 | 64.8 | 27 | 13.6 | 86 | 42.8 | 22 | 10.9 |
| **Musculoskeletal and connective tissue disorders** | 2 | 1.0 | 1 | 0.5 | 2 | 1.0 | 1 | 0.5 |
| Fistula/perforation | 2 | 1.0 | 1 | 0.5 | 2 | 1.0 | 1 | 0.5 |
| **Nervous system disorders** | 47 | 23.6 |  |  | 49 | 24.4 | 3 | 1.5 |
| Neurotoxicity, motor | 5 | 2.5 |  |  | 8 | 4.0 | 2 | 1.0 |
| Neurotoxicity, sensory | 45 | 22.6 |  |  | 46 | 22.9 | 1 | 0.5 |
| **Nephrotoxicity** | 29 | 14.6 | 1 | 0.5 | 51 | 25.4 | 4 | 2.0 |
| **Other** | 140 | 70.4 | 31 | 15.6 | 136 | 67.7 | 22 | 10.9 |
| **Psychiatric disorder** | 30 | 15.1 | 1 | 0.5 | 32 | 15.9 | 1 | 0.5 |
| **Skin and subcutaneous tissue disorders** | 176 | 88.4 | 60 | 30.2 | 119 | 59.2 | 9 | 4.5 |
| Alopecia | 57 | 28.6 | 1 | 0.5 | 81 | 40.3 | 3 | 1.5 |
| Dry skin | 101 | 50.8 | 4 | 2.0 | 37 | 18.4 | 1 | 0.5 |
| Hair disorder | 2 | 1.0 |  |  |  |  |  |  |
| Hand-foot-syndrome | 54 | 27.1 | 9 | 4.5 | 30 | 14.9 | 2 | 1.0 |
| Nail changes | 76 | 38.2 | 14 | 7.0 | 20 | 10.0 |  |  |
| Pruritus/itching | 66 | 33.2 | 2 | 1.0 | 22 | 10.9 | 1 | 0.5 |
| Rash/acne, acneiform | 156 | 78.4 | 36 | 18.1 | 19 | 9.5 |  |  |
| Rash/desquamation | 74 | 37.2 | 15 | 7.5 | 24 | 11.9 | 2 | 1.0 |
| Skin disorder, other | 35 | 17.6 | 4 | 2.0 | 14 | 7.0 |  |  |
| **Vascular disorders** | 71 | 35.7 | 32 | 16.1 | 101 | 50.2 | 38 | 18.9 |
| Hypertension | 49 | 24.6 | 14 | 7.0 | 75 | 37.3 | 14 | 7.0 |
| Thromboembolic event | 31 | 15.6 | 22 | 11.1 | 36 | 17.9 | 25 | 12.4 |
| **Toxicities of special relevance to cetuximab** |  |  |  |  |  |  |  |  |
| Acneiform exanthema | 156 | 78.4 | 36 | 18.1 | 19 | 9.5 |  |  |
| Desquamation | 74 | 37.2 | 15 | 7.5 | 24 | 11.9 | 2 | 1.0 |
| Paronychia | 76 | 38.2 | 14 | 7.0 | 20 | 10.0 |  |  |
| Infusion-related allergic reaction | 2 | 1.0 | 1 | 0.5 | 1 | 0.5 |  |  |
| Hypocalcaemia | 55 | 27.6 | 8 | 4.0 | 31 | 15.4 | 5 | 2.5 |
| Hypomagnesaemia | 126 | 63.3 | 9 | 4.5 | 60 | 29.9 | 14 | 0.7 |
| **Toxicities of special relevance to bevacizumab** |  |  |  |  |  |  |  |  |
| Hypertension | 49 | 24.6 | 14 | 7.0 | 75 | 37.3 | 14 | 7.0 |
| Proteinuria | 5 | 2.5 |  |  | 4 | 2.0 | 1 | 0.5 |
| Bleeding | 45 | 22.6 | 2 | 1.0 | 60 | 29.9 | 1 | 0.5 |
| Fistulae / perforation | 2 | 1.0 | 1 | 0.5 | 2 | 1.0 | 1 | 0.5 |
| Wound healing problems | 4 | 2.0 |  |  | 1 | 0.5 |  |  |
| Thromboembolic event | 31 | 15.6 | 22 | 11.1 | 36 | 17.9 | 25 | 12.4 |

GI, gastrointestinal; GU, genitourinary.

## **Table S.4. Efficacy in the *RAS* wild-type population (N=400)**

|  | FOLFIRI plus cetuximab (*N*=199) | FOLFIRI plus bevacizumab (*N*=201) | OR / HR (95% CI) | *P* value |
| --- | --- | --- | --- | --- |
| All patients |  |  |  |  |
| ORR, n (%)  [95% CI] | 131 (66) [59–72] | 118 (59) [52–66] | 1.36* (0.90–2.03) | 0.15 |
| Early tumour shrinkage, n (%) | 108/158 (68) | 85/174 (49) | 2.26* (1.45–3.54) | **0.0004** |
| Median depth of response, % (*n*=332) | 49 | 32 | NA | **<0.0001** |
| Median PFS, months  (95% CI) | 10 (9–12) | 10 (10–12) | 0.96^†^ (0.79–1.18) | 0.71 |
| Median OS, months  (95% CI) | 31 (25–36) | 26 (23–29) | 0.76^†^ (0.62–0.94) | **0.012** |
| Left-sided primary tumour | (*N*=158) | (*N* =149) |  |  |
| ORR, n (%)  [95% CI] | 109 (69) (61–76) | 92 (62) (53–70) |  |  |
| Early tumour shrinkage, n (%) | 89/126 (71) | 67/133 (50) |  |  |
| Median depth of response, % (*n*=259) | 50 | 33 |  |  |
| Median PFS, months  (95% CI) | 11 (10–12) | 11 (10–12) | 0.90^†^ (0.71–1.14) | 0.38 |
| Median OS, months  (95% CI) | 36 (30–41) | 28 (25–31) | 0.70^†^  (0.55–0.89) | **0.004** |
| Right-sided primary tumour | (*N*=38) | (*N*=50) |  |  |
| ORR, n (%)  [95% CI] | 20 (53) (36–69) | 25 (50) (36–65) | 1.11* (0.48–2.59) | 0.83 |
| Early tumour shrinkage, n (%) | 17/30 (57) | 16/39 (41) |  |  |
| Median depth of response, % (*n*=69) | 38 | 29 |  |  |
| Median PFS, months  (95% CI) | 8 (6–9) | 9 (7–12) | 1.41^†^ (0.91–2.19) | 0.13 |
| Median OS, months  (95% CI) | 18 (12–25) | 23 (19–26) | 1.27^†^ (0.81–1.98) | 0.29 |

* Odds ratio; † Hazard ratio.
CI, confidence interval; FOLFIRI, fluorouracil, folinic acid, and irinotecan; HR, hazard ratio; NA, not applicable; OR, odds ratio; ORR, objective response rate; OS, overall survival; PFS, progression-free survival.

## **Table S.5. Best overall response to treatment in the *RAS* wild-type and per-protocol populations**

| Response, n (%) | FOLFIRI plus  cetuximab | FOLFIRI plus bevacizumab | *P* value |
| --- | --- | --- | --- |
| *RAS* wild-type population (*N=*400) | (*N*=199) | (*N*=201) |  |
| Objective response: | 131 (66) | 118 (59) | 0.150 |
| Complete response | 8 (4) | 2 (1) |  |
| Partial response | 123 (62) | 116 (58) |  |
| Stable disease | 30 (15) | 56 (28) |  |
| Progressive disease | 9 (5) | 9 (5) |  |
| Not evaluable | 29 (15) | 18 (9) |  |
| Disease control rate* | 161 (81) | 174 (87) | 0.137 |
| Per-protocol population (*N=*352) | (*N*=169) | (*N*=183) |  |
| Objective response: | 130 (77) | 118 (65) | 0.014 |
| Complete response | 8 (5) | 2 (1) |  |
| Partial response | 122 (72) | 116 (63) |  |
| Stable disease | 30 (18) | 56 (31) |  |
| Progressive disease | 9 (5) | 9 (5) |  |
| Disease control rate* | 160 (95) | 174 (95) | >0.999 |

* Includes complete response, partial response and stable disease.
FOLFIRI, fluorouracil, folinic acid, and irinotecan.

**Table S.6 Early tumour shrinkage - by CMS-type**

**Population: Safety set, RAS wild-type, patient in analysis of central CT-review, CMS-group known, N=258**

| **Treatment arm / CMS-group** | | **Early tumour shrinkage** | | | | **Total** |
| --- | --- | --- | --- | --- | --- | --- |
|  |  | **No (tumor shrinkage > -20%)** | | **Yes (tumor shrinkage ≤ -20%)** | |  |
|  |  | **N** | **%** | **N** | **%** | **N** |
| FOLFIRI + cetuximab | 1 | 8 | 47.1 | 9 | 52.9 | 17 |
|  | 2 | 8 | 18.2 | 36 | 81.8 | 44 |
|  | 3 | 8 | 47.1 | 9 | 52.9 | 17 |
|  | 4 | 15 | 36.6 | 26 | 63.4 | 41 |
|  | Total | 39 | 32.8 | 80 | 67.2 | 119 |
| FOLFIRI + bevacizumab | 1 | 12 | 60.0 | 8 | 40.0 | 20 |
|  | 2 | 27 | 42.9 | 36 | 57.1 | 63 |
|  | 3 | 8 | 66.7 | 4 | 33.3 | 12 |
|  | 4 | 27 | 61.4 | 17 | 38.6 | 44 |
|  | Total | 74 | 53.2 | 65 | 46.8 | 139 |
| Overall | 1 | 20 | 54.1 | 17 | 45.9 | 37 |
|  | 2 | 35 | 32.7 | 72 | 67.3 | 107 |
|  | 3 | 16 | 55.2 | 13 | 44.8 | 29 |
|  | 4 | 42 | 49.4 | 43 | 50.6 | 85 |
|  | Total | 113 | 43.8 | 145 | 56.2 | 258 |

**Supplementary Table S7**

**Baseline characteristics of patients surviving 3, 4, and 5 years**

| **Parameter** | **FOLFIRI + Cetuximab**  **3-y-OS**  **N=81** | **FOLFIRI + Bev**  **3-y-OS**  **N=60** | **FOLFIRI + Cetuximab**  **4-y-OS**  **N=48** | **FOLFIRI + Bev**  **4-y-OS**  **N=31** | **FOLFIRI + Cetuximab**  **5-y-OS**  **N=25** | **FOLFIRI + Bev**  **5-y-OS**  **N=13** |
| --- | --- | --- | --- | --- | --- | --- |
| Age – median (years) | 63 | 63 | 60 | 63 | 60 | 65 |
| Age - min (years) | 41 | 33 | 42 | 46 | 42 | 47 |
| Age - max (years) | 76 | 75 | 76 | 75 | 76 | 69 |
| Age > 65 years (%) | 45.7 | 40.0 | 35.4 | 45.2 | 28.0 | 46.2 |
|  |  |  |  |  |  |  |
| Female (%) | 25.9 | 35.0 | 22.9 | 35.5 | 20.0 | 30.8 |
|  |  |  |  |  |  |  |
| ECOG 0 (%) | 58.0 | 65.0 | 64.6 | 67.7 | 64.0 | 69.2 |
| ECOG 1 (%) | 40.7 | 33.3 | 33.3 | 29.0 | 32.0 | 30.8 |
| ECOG 2 (%) | 1.2 | 1.7 | 2.1 | 3.2 | 4.0 | - |
|  |  |  |  |  |  |  |
| Colon (%) | 53.1 | 65.0 | 47.9 | 67.7 | 48.0 | 69.2 |
| Rectum (%) | 44.4 | 33.3 | 52.1 | 29.0 | 52.0 | 30.8 |
| Colon and rectum (%) | 2.5 | 1.7 | - | 3.2 | - | - |
|  |  |  |  |  |  |  |
| Left-sided (%) | 91.4 | 81.7 | 95.8 | 80.6 | 92.0 | 92.3 |
| Right-sided (%) | 7.4 | 18.3 | 4.2 | 19.4 | 8.0 | 7.7 |
| Both-sided or unknown (%) | 1.2 | - | - | - | - | - |
|  |  |  |  |  |  |  |
| Metastatic sites: 1 (%) | 48.1 | 53.3 | 52.1 | 58.1 | 60.0 | 84.6 |
| Metastatic sites: 2 (%) | 34.6 | 26.7 | 33.3 | 16.1 | 24.0 | - |
| Metastatic sites: ≥ 3 (%) | 16.0 | 18.3 | 14.6 | 22.6 | 16.0 | 7.7 |
| Metastatic sites: unknown (%) | 1.2 | 1.7 | - | 3.2 | - | 7.7 |
|  |  |  |  |  |  |  |
| Liver only (%) | 43.2 | 40.0 | 43.8 | 38.7 | 56.0 | 69.2 |
| Lung only (%) | 2.5 | 10.0 | 4.2 | 16.1 | - | 15.4 |
|  |  |  |  |  |  |  |
| Primary tumour resection (%) | 87.7 | 90.0 | 83.3 | 93.5 | 80.0 | 92.3 |
| Adjuvant chemotherapy (%) | 19.8 | 23.3 | 29.2 | 32.3 | 20.0 | 15.4 |

## **Figure S.1. Distribution of patients included in the RAS wild-type (N=400, top) and per-protocol (N=352, bottom) analyses**


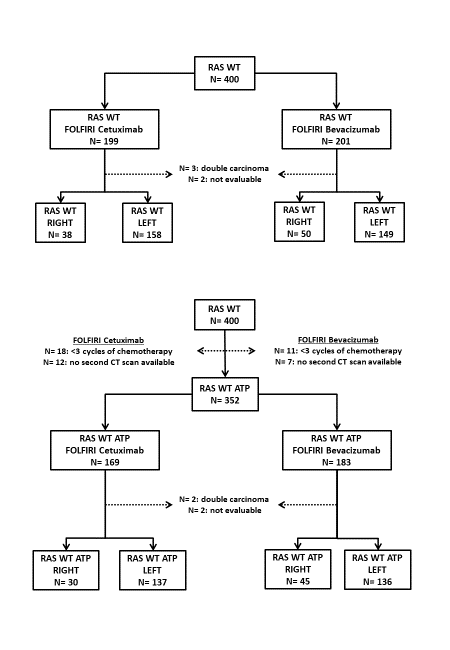


ATP, according to protocol; WT, wild-type.
Left and right refer to the location of the primary tumour.

## **Figure S.2. Primary tumour location and survival in the *RAS* wild-type population**

Left-sided tumours (*N*=307): **a.** Progression-free survival, **b.** Overall survival; Right-sided tumours (*N*=88): **c.** Progression-free survival, **d.** Overall survival.

**(a)**


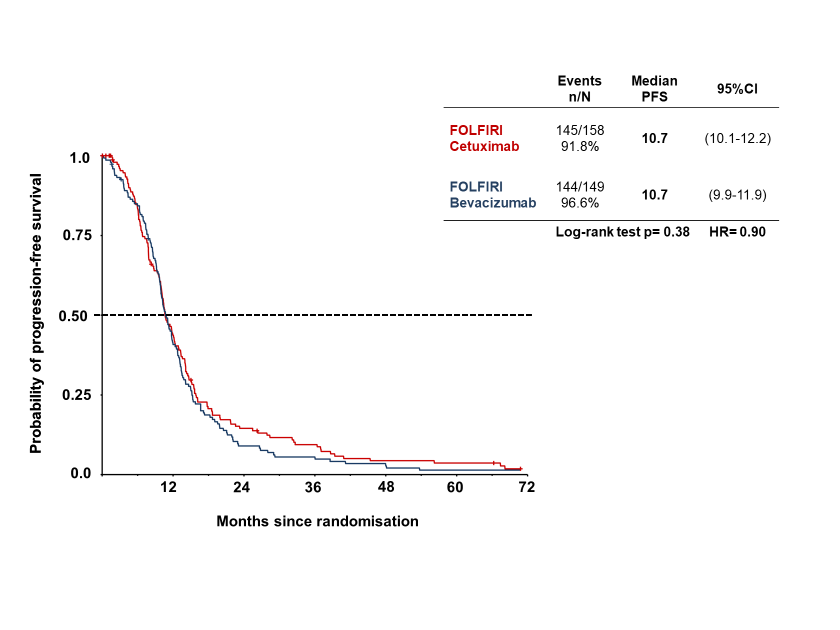


**(b)**


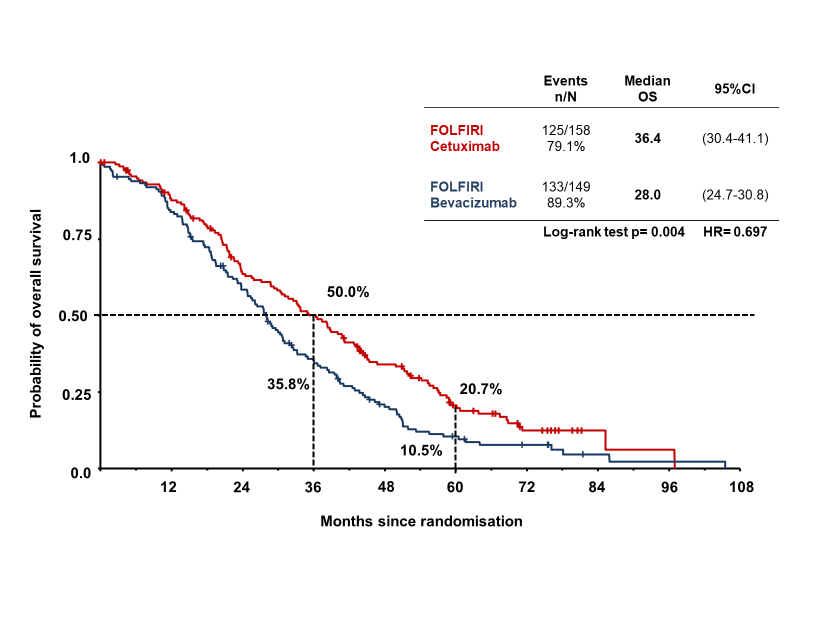


**(c)**


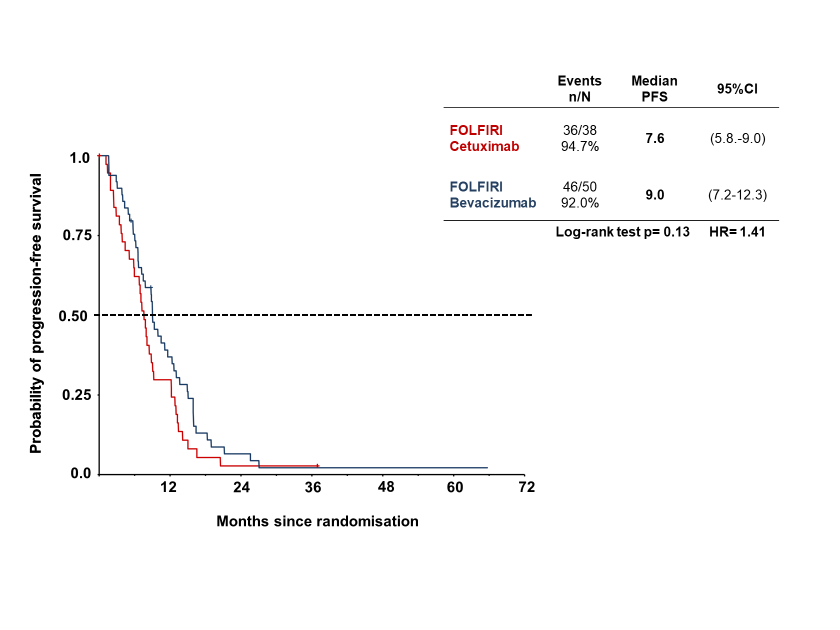


**(d)**


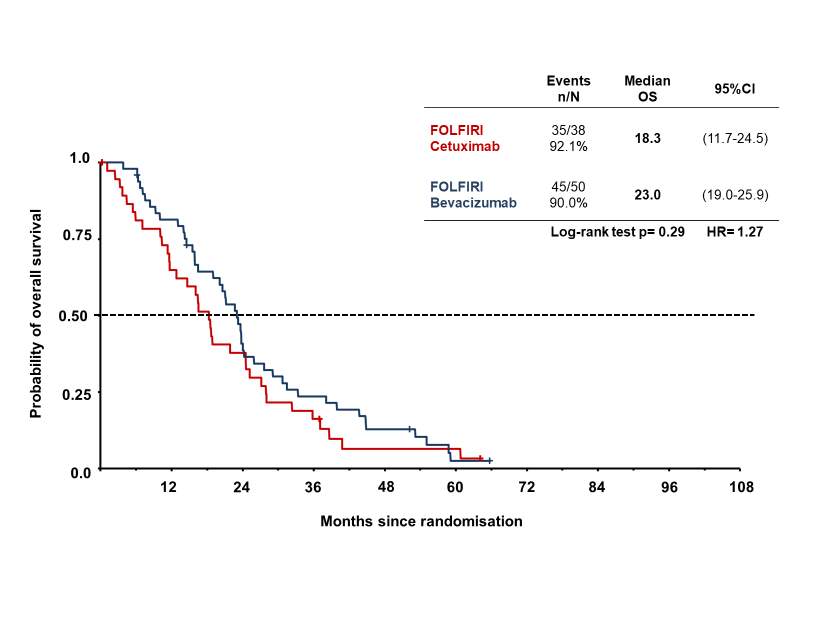

Supplement: Supplementary file 1 — Supplementary Data Set [file 41416_2020_1140_MOESM1_ESM.docx]
